# Age-Related Changes in the Nasolabial Angle and Anterior Nasal Spine: A Three-Dimensional Morphometric Study Using Skull Computed Tomography (CT) Scans

**DOI:** 10.7759/cureus.105992

**Published:** 2026-03-27

**Authors:** Isabela L de Paula, Alexandre R Freire, Beatriz C Ferreira-Pileggi, Camila C Furlan, Felippe B Prado, Ana Cláudia Rossi

**Affiliations:** 1 Biosciences, Piracicaba Dental School, University of Campinas (UNICAMP), Piracicaba, BRA

**Keywords:** aging, anatomy, anterior nasal spine, morphometric, nasolabial angle

## Abstract

Introduction: The anterior nasal spine (ANS) is a thin bony structure located in the maxilla that plays a crucial role in supporting the nasal apex and upper lip, serving as an important reference point in dental and surgical procedures, as well as in craniofacial analyses. Age-related changes, such as bone remodeling, can influence its morphology and, consequently, modify the nasolabial angle, a relevant parameter for assessing aesthetic and functional changes in the face. The objective of this study was to investigate possible changes in the nasolabial angle with advancing age through three-dimensional morphometric analysis of computed tomography scans of human skulls.

Methods: The sample consisted of 93 CT scans. Based on age, the individuals were classified into three groups: 18-44 years (young), 45-64 years (middle-aged), and ≥65 years (elderly). The images were segmented using Mimics 18.0 software, allowing three-dimensional reconstruction of the ANS and measurement of the nasolabial angle from three specific anatomical landmarks. Statistical analysis was performed using ANOVA, followed by a Šídák multiple comparisons test, adopting a 5% significance level.

Results: The results demonstrated that the nasolabial angle presented significant changes from the young male group vs. the middle-aged female group, tending to become more closed with aging. Furthermore, morphological variations of the ANS (single, bifid, or double) were identified, highlighting its anatomical diversity.

Conclusion: It is concluded that the ANS undergoes structural modifications throughout adulthood, as reflected in the nasolabial angle, and that it is a relevant landmark in studies of facial aging, in addition to its clinical value in dental and surgical procedures.

## Introduction

The anterior nasal spine (ANS) is a thin bony protrusion of the maxilla, located in the median sagittal plane at the base of the piriform aperture, and, radiographically, is a V-shaped radiopaque area. This anatomical structure is extremely important in determining the morphology of the face in several specialties of dentistry since it provides support to the tip of the nose and ensures the projection of structures such as the nose and upper lip [1].

Thus, the ANS is an important anatomical landmark for surgeries involving the maxillofacial region and dental procedures. In dentistry, for example, the ANS is a reliable reference point for determining the occlusal vertical dimension in individuals who have experienced loss of this vertical dimension [2].

The ANS is a prominent anatomical feature of the maxilla, positioned at the upper anterior end of the intermaxillary suture. It represents a bone structure that occurs exclusively in humans, unlike in other primates and mammals [3]. The anteroinferior border of the nasal septum is connected to the ANS and is considered an important factor in defining facial morphology, particularly in providing support to the nose and upper lip [3]. Furthermore, the ANS provides support to the nasal septum and contributes to the preservation of the nasolabial angle, defined as the angle formed between the lower border of the nasal septum and the craniometric point A [3]. If this structure is underdeveloped or retropositioned, a drooping nasal tip, typically related to aging in the nasal region, may be observed as a secondary feature [4].

Jeon et al. [5] stated that the aging process causes changes in the facial skeleton, as continuous bone remodeling factors, such as expansion, augmentation, and resorption, can also influence the appearance of adjacent soft tissues, altering craniofacial morphology and, consequently, facial anatomy. The midface region, where the ANS is located, tends to undergo retrusion due to uneven bone resorption, which may limit the beneficial potential of aesthetic procedures aimed at rejuvenation.

Although the ANS is an anatomical reference point in dentistry, it has not been the focus of scientific research. With the advent of Orofacial Harmonization as a specialty in dentistry, it is now known that, among the various treatment modalities for facial rejuvenation, increasing the volume of the mid-face region is considered one of the safest ways to maintain or restore facial youthfulness, given that the skeleton of the mid-face region undergoes more radical changes with aging [6], and ANS can likely accompany these midface skeletal changes [7].

The assessment of age-related variations in the nasolabial angle defined by the ANS may constitute a relevant parameter for craniofacial morphological analysis. The ANS functions as a stable anatomical landmark whose dimensional and positional alterations can reflect structural changes associated with the aging process in the nasal region. Thus, the aim of the present study was to investigate whether the nasolabial angle can change with age using three-dimensional morphometric analysis on computed tomography scans of human skulls.

## Materials and methods

Sample

The sample consisted of 93 computed tomography scans of cataloged individuals belonging to the "Osteological and Tomographic Prof. Dr. Eduardo Daruge" Biobank of Piracicaba Dental School at the University of Campinas (UNICAMP). The individuals belonged to a contemporary population from southeastern Brazil, and information such as sex and age was identified from death certificates. The tomographic images were acquired using an Asteion Multislice 4 CT System (Toshiba Medical Systems Corporation, Japan) using the cranial protocol: 100 mA, 120 kV, with 1 mm slices. 

The sample size was defined based on the availability of specimens from the osteological collection, characterizing a convenience sample. The groups were inherently unbalanced due to the limited number of available skulls in specific sex and age categories.

Computed tomography scans (CT scans) of preserved skulls without visible deformities, fractures, or any pathological or surgical alterations were included. CT scans of skulls from syndromic individuals or those presenting anatomical abnormalities in the region of interest were excluded, as well as scans showing implants, plates, screws, or other metallic artifacts near this area.

The CT scans with age information were divided into three groups according to the age categories defined by Shaw and Kahn [6]: young (25 to 44 years old), middle-aged (45 to 64 years old), and elderly (≥65 years old).

Segmentation of tomographic images

The software Mimics 18.0 (Materialise, NV, Belgium) was used to segment the images of each CT scan. Segmentation consisted of selecting the pixels of the bone structure in each CT slice. This selection was defined by evaluating a grayscale threshold that was applied to select voxels with values within a specific range corresponding to the anatomical structure of the ANS. The 3D reconstruction was performed to enable visualization of this component, and the three-dimensional surface was exported in virtual stereolithography (.STL) for surface evaluation.

Three-dimensional morphometric analysis

**Figure 1:**
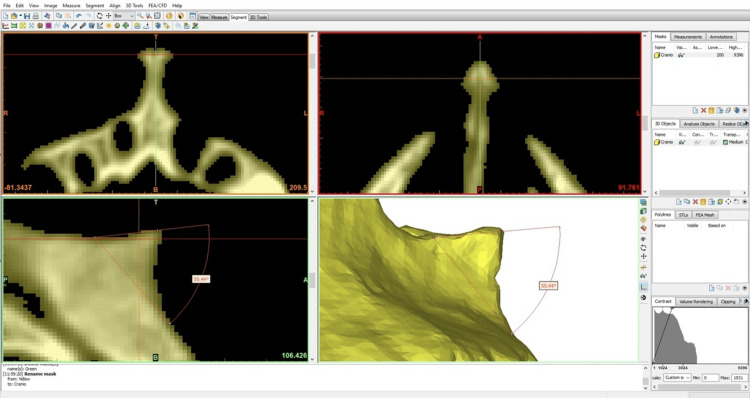
Lateral view of a three-dimensional reconstruction derived from a computed tomography scan of the human skull, illustrating the anterior nasal spine (ANS) and the nasolabial angle, identified using a measurement tool (in degrees). Layout of the Mimics 18.0 software (Materialise, NV, Belgium). Statistical analyses included the Shapiro–Wilk test for normality and two-way analysis of variance (ANOVA) followed by Šídák's post hoc test, adopting a significance level of 5%.

To obtain the nasolabial angle, Mimics 18.0 software (Materialise, NV, Belgium) was used to generate the three-dimensional surface of the already segmented tomographic images. Then, an angle assessment tool (in degrees) was applied to the region of interest, where three points were marked to generate the angle value. The marked points were: 1) tip of the ANS; 2) base (contour) of the piriform aperture; 3) craniometric point A (an arbitrary point that delimits the alveolar part with the body of the maxilla, located at the most depressed point of the maxillary contour) (Figure 1).

Reliability and calibration

All measurements were performed by a single trained examiner using a standardized protocol. Measurements were obtained through digital/computational analysis, reducing operator-dependent variability.

To assess intra-examiner consistency, 20% of the sample was randomly selected and re-measured after a two-week interval. The repeated measurements showed minimal variation, indicating satisfactory reproducibility.

Data analysis

Data were tabulated in Microsoft Office Excel®, and statistical analysis was performed using GraphPad Prism v.10 (San Diego, CA, USA), adopting a significance level of 5%. Descriptive statistics were expressed as mean and standard deviation (SD) for continuous variables and percentages (%) for categorical variables. Data normality was assessed using the Shapiro-Wilk test, and homogeneity of variances was evaluated using Levene’s test. For comparisons of nasolabial angle values, a two-way analysis of variance (ANOVA) was conducted to assess the main effects of sex and age, as well as their interaction. When significant effects were identified, multiple comparisons were performed using Šídák’s post hoc test. Effect size was calculated using eta squared (η²).

## Results

Of the 93 computed tomography scans of skulls evaluated, 36 are female, of which 17 (47.222%) are elderly individuals, 10 (27.778%) are young individuals and 9 (25%) are middle-aged individuals; 57 are male, of which 15 (26.316%) are elderly, 16 (28.070%) are young individuals and 26 (45.614%) are middle-aged.

In the present study, anatomical variations of the ANS were observed. In the elderly female group, 16 (44.444%) ANS were single, and 1 (2.778%) was bifid. In the young female group, 8 (22.222%) were single, 1 (2.778%) was bifid, and 1 (2.778%) was double. In the middle-aged female group, 6 (16.667%) were single, 2 (5.556%) were bifid, and 1 (2.778%) was double. In the elderly male group, 10 (17.544%) were single, 4 (7.018%) were bifid, and 1 (1.754%) was double. In the young male group, 11 (19.298%) were single, 3 (5.263%) were bifid, and 2 (3.509%) were double. In the middle-aged male group, 20 (35.088%) were single, 3 (5.263%) were bifid, and 3 (5.263%) were double (Table 1).

**Table 1:** Frequency and percentage distribution of anterior nasal spine morphologies (simple, bifid, and double) observed in the analyzed sample. Percentages were calculated separately within each sex, using n = 36 for females and n = 57 for males.

Age Group	Female – Single (n, %)	Female – Bifid (n, %)	Female – Double (n, %)	Male – Single (n, %)	Male – Bifid (n, %)	Male – Double (n, %)
Young	8 (22.222%)	1 (2.778%)	1 (2.778%)	11 (19.298%)	3 (5.263%)	2 (3.509%)
Middle-aged	6 (16.667%)	2 (5.556%)	1 (2.778%)	20 (35.088%)	3 (5.263%)	3 (5.263%)
Elderly	16 (44.444%)	1 (2.778%)	–	10 (17.544%)	4 (7.018%)	1 (1.754%)

The angles of the ANS were measured for all individuals. The distribution of nasolabial angle values was assessed using the Shapiro-Wilk test, confirming the normal distribution of the data. The two-way ANOVA revealed a statistically significant effect of age on the ANS angles (p = 0.0182). However, no significant differences were observed for sex (p = 0.0882) or for the interaction between age and sex (p = 0.0710). The predicted means were 61.80 for males and 57.24 for females, with a non-significant difference of 4.56 (95% CI = −0.70 to 9.82) (Table 2).

**Table 2:** Two-way ANOVA results for the nasolabial angle (°) in individuals with a single ANS, according to sex and age group. The analysis revealed a significant effect of age (p = 0.0182), with no significant effects of sex (p = 0.0882) or the interaction between age and sex (p = 0.0710). F = test statistic; DFn/DFd = degrees of freedom η²: effect size. Statistically significant difference (p < 0.05).

Factor	F (DFn, DFd)	P-value	η^2^
Age	F(2, 65) = 4.262	0.0182*	0.104
Sex	F(1, 65) = 2.996	0.0882	0.037
Age x Sex Interaction	F(2, 65) = 2.755	0.0710	0.067

**Figure 2:**
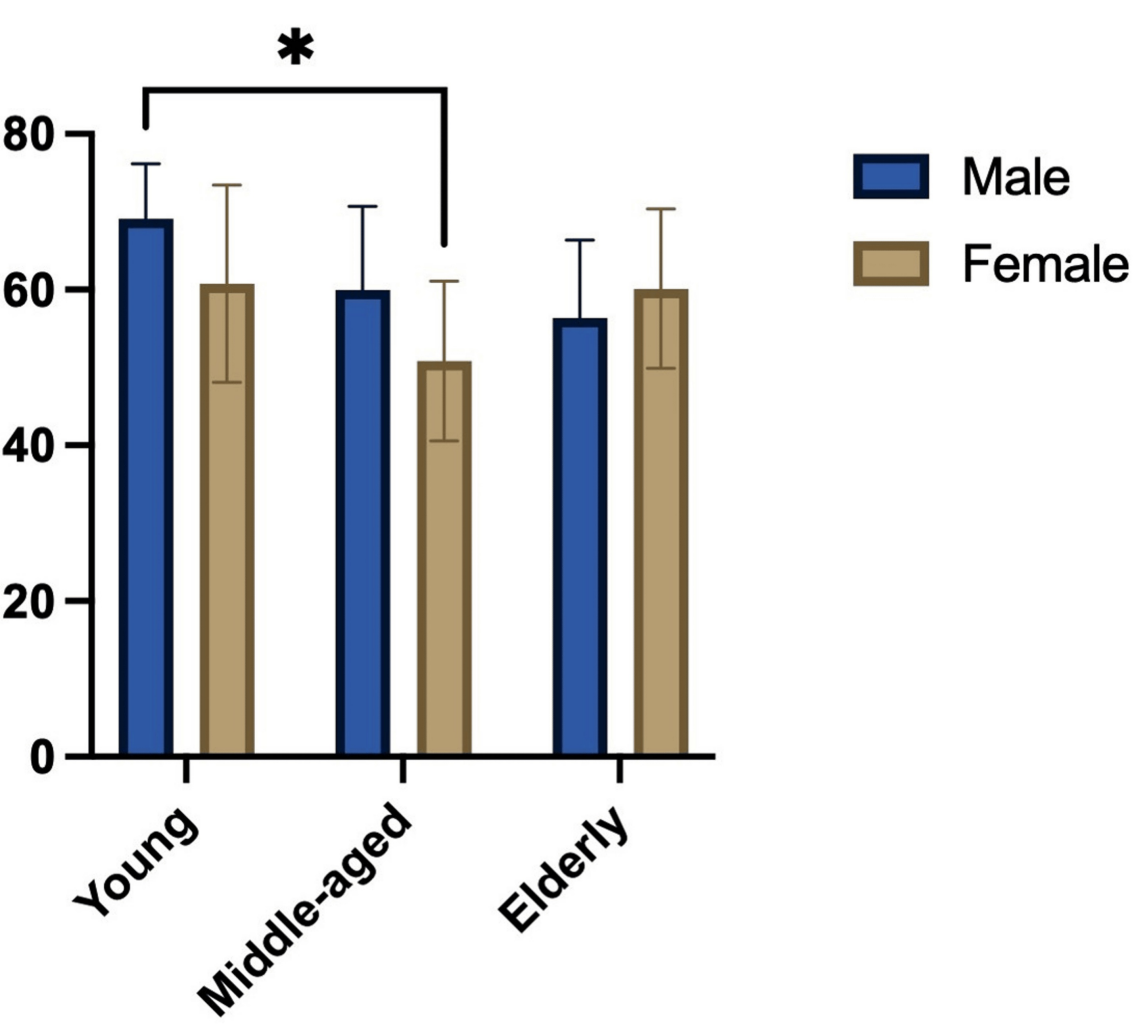
Graph showing the nasolabial angle (°) in the evaluated groups, representing the results of the multiple comparisons analysis. Data are presented as mean ± standard deviation. Statistical analysis was performed using two-way ANOVA followed by Šídák’s multiple comparisons test. A statistically significant difference was observed between young males and middle-aged females (p = 0.0122). *Statistically significant difference (p < 0.05)

Post hoc multiple comparisons using Šídák’s test revealed statistically significant differences between young male vs. middle-aged female (p = 0.0122). The remaining comparisons showed no statistically significant differences. These results are summarized in Table 3 and illustrated in Figure 2, which also presents the group means and variability.

**Table 3:** Post hoc pairwise comparisons of the measured angle (°) among sex and age groups using Šídák’s multiple comparisons test following two-way ANOVA. Values represent the mean difference between groups (°) and the adjusted p-value for multiple comparisons. *Statistically significant difference (p < 0.05)

Comparison	Mean Difference	Adjusted p-value
Young Male vs. Young Female	8.32	0.7371
Young Male vs. Middle-aged Male	9.12	0.2692
Young Male vs. Middle-aged Female	18.25	0.0122*
Young Male vs. Elderly Male	12.74	0.0851
Young Male vs. Elderly Female	8.97	0.3552
Young Female vs. Middle-aged Male	0.80	>0.9999
Young Female vs. Middle-aged Female	9.93	0.7010
Young Female vs. Elderly Male	4.42	0.9989
Young Female vs. Elderly Female	0.65	>0.9999
Middle-aged Male vs. Middle-aged Female	9.13	0.6044
Middle-aged Male vs. Elderly Male	3.62	0.9989
Middle-aged Male vs. Elderly Female	-0.15	>0.9999
Middle-aged Female vs. Elderly Male	-5.51	0.9954
Middle-aged Female vs. Elderly Female	-9.28	0.6227
Elderly Male vs. Elderly Female	-3.77	0.9989

## Discussion

The ANS is a thin bony protrusion formed by the union of the two maxillae in the midsagittal plane. This anatomical structure is located at the base of the piriform aperture in the midsagittal plane and is radiographically visible as a V-shaped radiopaque area. Essential in determining facial morphology, the ANS supports the apex of the nose and the upper lip and is a crucial anatomical landmark in maxillofacial surgery and dental procedures, for example, by serving as a reference and aiding in determining the occlusal vertical dimension [1,2].

Anatomically unique in humans, the ANS connects to the anteroinferior border of the nasal septum, influencing facial shape and the support of the nose and upper lip [3], a factor that maintains the nasolabial angle. Changes in the development of the ANS, when underdeveloped or retro-positioned, can lead to a drooping nasal apex, a characteristic of aging [4]. Jeon et al. [5] stated that continuous bone remodeling factors, such as expansion, augmentation, and resorption, cause changes in the facial skeleton and can also influence the appearance of adjacent template tissues, modifying craniofacial morphology and, consequently, local facial anatomy. The midface region, where the ANS is located, tends to undergo retrusion due to irregular bone resorption. This was confirmed by Tonelli [8] through a morphometric analysis of three-dimensional models. The author observed a reduction in the projection of skeletal structures and their overlying structures, such as ligaments and muscle insertions, particularly in the piriform aperture, the orbital cavity, and the maxilla. Uneven bone resorption can limit the beneficial potential of aesthetic procedures aimed at rejuvenation. Thus, bone remodeling over the years can also alter the points that determine the nasolabial angle, causing changes in this measurement with age.

In the present study, it was found that the nasolabial angle, determined by the points 1) tip of the ANS; 2) base (contour) of the piriform aperture; 3) craniometric point. In the present study, it was found that the nasolabial angle was significantly different only between young males and middle-aged females, with a tendency toward a more closed angle in the latter group. No other pairwise comparisons showed statistically significant differences, suggesting a tendency toward a more closed angle with aging, although this pattern was not consistently observed across all group comparisons. Holton et al. [9] state that the nasal septum influences the anteroposterior dimensions of the facial skeleton during the early stages of development, which also affects the projection of the ANS. Through CT scans, the researchers concluded that individuals with a smaller nasal septum present a decrease in external nasal projection, characterized by a flatter nasal bridge and reduced prominence of the ANS, with the opposite also being true. This factor, in turn, also interferes with the determining points for the formation of the nasolabial angle, since it characterizes the establishment of this measurement in younger individuals, who will subsequently undergo a prolonged and more accentuated period of bone remodeling, with a tendency to form a more closed angle, as we observed in the results of this work among young and middle-aged individuals.

Davidson et al. [10] evaluated the metric data of the ANS using cone beam computed tomography (CBCT) images, analyzing the sagittal and axial planes, where the structure was most clearly visualized. The authors obtained both linear and angular measurements. Davidson et al. [10], when analyzing metric data related to the ANS, concluded that the minimal differences related to the length and angles resulting from the measurements of this structure can also be attributed to sexual dimorphism, also justifying the differences, although not significant, observed between the female and male sexes in this research.

In the present study, anatomical variations of the ANS were also identified, with morphologies that can be classified as single, bifid, or double. The existing literature reveals a scarcity of available data to determine the prevalence of these anatomical variations in different populations, which prevents comparisons regarding their incidence. However, given the ANS's function in supporting the apex of the nose, these morphologies have been mentioned in studies related to rhinoplasties and fractures that directly affect the positioning of the nasal septum [11]. You et al. [12] observed that ANS fractures resulting from maxillofacial trauma are not uncommon (22%), especially in single and double morphologies, but that the diagnosis of these fractures often goes unnoticed (95.45%) due to a lack of attention to this anatomical structure.

Therefore, based on the findings of this study in a Brazilian population, detailed knowledge of the anterior nasal spine (ANS), its morphological variations, and its relationships with adjacent anatomical structures is essential for understanding the nasolabial angle and its potential changes with aging. The present findings provide relevant anatomical reference values according to sex and age groups, which may assist clinicians in orthodontic diagnosis, facial aesthetic evaluation, and treatment planning. Furthermore, understanding these morphological differences may contribute to more individualized approaches in maxillofacial and reconstructive procedures, potentially improving functional and aesthetic outcomes. These data also serve as a reference for clinical and surgical procedures, supporting diagnostic decision-making and highlighting the need for further studies in this field.

This study has some limitations that should be considered. The retrospective design and the use of a limited sample of CT scans may restrict the generalization of the findings. In addition, the studied population represents a genetically admixed population, which may influence anatomical characteristics such as ANS morphology and should be considered when interpreting the results. Therefore, further studies with larger and more diverse samples are recommended to further explore these findings.

## Conclusions

In conclusion, the ANS exhibits morphological variability and undergoes age-related changes that influence the nasolabial angle. In the present study, ANS morphologies were classified as single, bifid, and double, with the single type being the most frequent. A significant difference in the nasolabial angle was observed only between young males and middle-aged females. Additionally, age showed a significant effect on the nasolabial angle, whereas sex did not. These findings highlight the importance of considering anatomical variations and age-related skeletal changes when evaluating midfacial aging, as well as the relevance of a comprehensive understanding of ANS morphology for accurate diagnosis, aesthetic assessment, and surgical planning in maxillofacial and dental procedures.
